# Investigating Association between Intraoperative Hypotension and Postoperative Neurocognitive Disorders in Non-Cardiac Surgery: A Comprehensive Review

**DOI:** 10.3390/jcm9103183

**Published:** 2020-09-30

**Authors:** Łukasz J. Krzych, Michał P. Pluta, Zbigniew Putowski, Marcelina Czok

**Affiliations:** 1 Department of Anaesthesiology and Intensive Care, Faculty of Medical Sciences in Katowice, Medical University of Silesia, 40752 Katowice, Poland; 2 Students’ Scientific Society, Department of Anaesthesiology and Intensive Care, Faculty of Medical Sciences in Katowice, Medical University of Silesia, 40752 Katowice, Poland; michal_p2@o2.pl (M.P.P.); putowski.zbigniew@gmail.com (Z.P.); mczok@poczta.fm (M.C.)

**Keywords:** hypotension, cognitive functioning, cognitive decline, delirium, neurocognitive complications, perioperative

## Abstract

Postoperative delirium (POD) and postoperative cognitive decline (deficit) (POCD) are related to a higher risk of postoperative complications and long-term disability. Pathophysiology of POD and POCD is complex, elusive and multifactorial. Intraoperative hypotension (IOH) constitutes a frequent and vital health hazard in the perioperative period. Unfortunately, there are no international recommendations in terms of diagnostics and treatment of neurocognitive complications which may arise from hypotension-related hypoperfusion. Therefore, we performed a comprehensive review of the literature evaluating the association between IOH and POD/POCD in the non-cardiac setting. We have concluded that available data are quite inconsistent and there is a paucity of high-quality evidence convincing that IOH is a risk factor for POD/POCD development. Considerable heterogeneity between studies is the major limitation to set up reliable recommendations regarding intraoperative blood pressure management to protect the brain against hypotension-related hypoperfusion. Further well-designed and effectively-performed research is needed to elucidate true impact of intraoperative blood pressure variations on postoperative cognitive functioning.

## 1. Introduction

Intraoperative hypotension (IOH) is frequent and multifactorial (Table 1) [1,2,3]. The Perioperative Quality Initiative (POQI) consensus statement on intraoperative blood pressure (BP), risks and outcomes for elective non-cardiac surgery has warned that there is increasing evidence that even brief durations of hypotension, regardless of its origin, are harmful [4]. The above-cited document is based on a comprehensive review of current international data. Although the evidence that IOH is associated with myocardial injury (MINS, myocardial injury in non-cardiac surgery), acute kidney injury (AKI) and death is clear, there is a paucity of data that ischemia-reperfusion injury due to hypotension may substantially affect the brain. The POQI experts considered several topics to be research priorities in the nearest future. Unfortunately, they gave no recommendations for further investigations in terms of neurocognitive complications which may arise from hypotension-related hypoperfusion.

According to recent recommendations, the term ‘perioperative neurocognitive disorders’ encompasses cognitive impairment identified in the preoperative or postoperative period. This includes cognitive decline diagnosed before operation (described as neurocognitive disorder); any form of acute event (postoperative delirium, POD) and cognitive decline diagnosed up to 30 days post-surgery (delayed neurocognitive recovery) and up to 12 months (postoperative neurocognitive disorder, POCD) [5]. Their pathophysiology is complex, elusive and likely multifactorial [6].

POD is defined as disturbance of consciousness with reduced ability to focus, sustain and shift attention, and awareness. An additional disturbance in cognition (memory deficit, disorientation, language, visuospatial ability or perception) appears [7]. It has an acute onset and fluctuating course. POD is defined as that which occurs in hospital up to 1 week post-procedure or until discharge (whichever occurs first). POD can be diagnosed by non-psychiatrists using accurate and validated bedside tools [8]. POCD affects global cognitive functions for several months after surgery and its diagnosis requires detailed neuropsychological testing.

POCD and POD are more frequent in vulnerable populations (Table 2 and Table 3). More to the point, they are often related to a higher risk of other postoperative complications and long-term disability [6,9,10,11]. The International Study of Postoperative Cognitive Dysfunction (ISPOCD) group has underlined that POCD is associated with reduced daily activities, stopping work earlier in life, increased dependency on social support and increased mortality [12,13].

As there is a clear paucity of data regarding this interesting issue, we aimed to investigate the association between IOH and POCD, or POD in non-cardiac surgery, basing on a comprehensive review of the literature. Due to explicit heterogeneity between publications and subsequent risk of bias we failed to prepare a systematic review with meta-analysis.

## 2. Methods

To avoid publication bias, we applied a structured systematic search strategy of all relevant publications in the field (Table 4).

**Figure 1:**
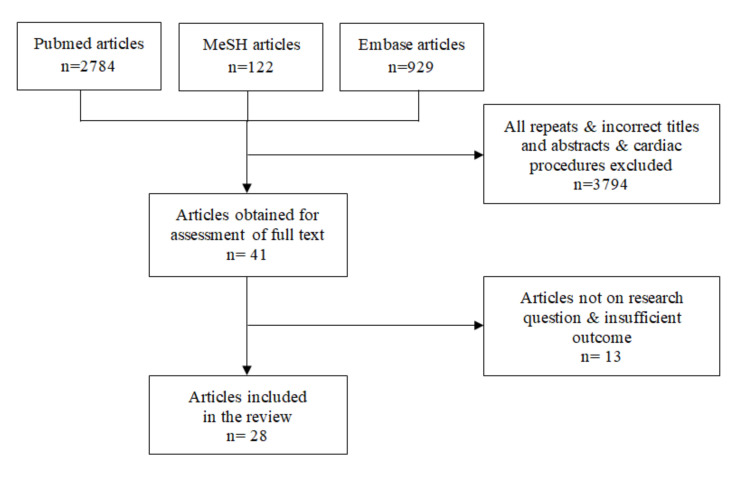
Flow chart of the screened and included papers.

This search strategy was prepared by an experienced librarian in cooperation with investigators. Basically, inclusions were established by using medical subject heading (MeSH) terms and key words, according to the study aim (*n* = 3835). Then, we excluded all repeats, papers unrelated to IOH, POCD, POD and those performed in a cardiac surgery setting (*n* = 3794). All remaining 41 papers were assessed individually by 4 independent investigators. This step included detailed verification of the results of each study and their relation to IOH, POCD and POD. Then, an investigators meeting was set up to discuss all the papers. The study was processed further if at least 3 of 4 adjudicators agreed to include the paper for review. If only two reviewers agreed to proceed the manuscript, the second assessment of the paper was performed by all investigators collectively (Figure 1).

## 3. Review

### 3.1. Postoperative Cognitive Decline (Deficit)

With the increasing number of non-cardiac surgeries performed worldwide, with over 300 million procedures annually, also in the high-risk and aging population, POCD could pose a significant threat to global health issues [26]. Surprisingly, there is a limited number of studies focused on assessing the role of intraoperative BP drop and the development of POCD, despite its clear pathophysiological association [27].

Between 1993 and 1995, a randomized controlled trial including 235 patients older than 50 years (mean age 72 years) undergoing total hip replacement with neuraxial anesthesia was performed [28]. Subjects were randomly assigned into two arms: ‘controlled IOH’ group, with a mean arterial pressure (MAP) of 45–55 mmHg or a group with 55–75 mmHg. The researchers found no significant differences between those two groups in terms of either early (i.e., 1 week after surgery) or late (i.e., 4 months after surgery) POCD. POCD was defined as a within-subject change in score for each of 10 neuropsychological tests over a 4-month period. It is vital to note that it is difficult to draw reliable conclusions based on the above-mentioned data. Although all subjects were hypotensive during surgery, no other adverse events were assessed, including MINS and AKI. Also, no control group (i.e., without hypotension) was applied to verify if MAP of 45–55 mmHg was safe to maintain adequate organ perfusion.

Moller et al. published the results of a multicenter study, in which 1218 surgical patients aged ≥ 60 years undergoing major abdominal, thoracic and orthopedic procedures under general anesthesia between 1994 and 1996, were tested for POCD after 1 week and 3 months after procedure using a battery of neuropsychological tests [12]. In their study, IOH was defined as an MAP drop of at least 60% from baseline value for at least 30 min. No statistical significance was observed in POCD occurrence between ‘hypotensive’ and ‘non-hypotensive’ subjects, at either 1 week (i.e., ‘early POCD’) or 3 months (‘late POCD’) postoperatively. Lack of association between hypotension and cognitive decline might have resulted from a unique definition of IOH, which was very strict and incomparable with other research cited in this review, and different from current recommendations of the POQI [4].

Brazilian researchers, in a study of 55 patients undergoing orthopedic, urologic, general or vascular surgeries in general (*n* = 28) or regional (*n* = 27) anesthesia, assessed the incidence and risk factors of POCD defined as Mini-Mental State Examination (MMSE) scores below 24/30 or by a decrease of 4 or more points during follow-up evaluations [29]. The incidence of IOH, defined as systolic BP decrease of more than 30% as compared to baseline values, lasting more than 5 min or needing vasopressor or inotropic support, did not differ between subjects who developed or did not develop POCD (*p* = 0.7). The study was underpowered (*n* = 55) and the results were not adjusted for a plethora of possible confounding factors.

In 2009, Yocum et al. published a post-hoc cohort analysis in which 24 hypertensive and 21 normotensive patients aged ≥40 years were screened for POCD after lumbar laminectomy or microdiscectomy [30]. A battery of 5 neuropsychometric tests was used for assessing cognitive performance preoperatively (baseline) at 1 day, and 1 month postoperatively. The blood pressure was recorded as a fraction of preoperative MAP (i.e., % of baseline MAP). The lowest fractional MAPs, together with several other factors (age, hypertension, years of education, duration of surgery, etc.) were put into multivariate analysis in which the sole presence of either hypertension or lowest fractional MAP was not associated with POCD. However, hypertension crossed with minimum MAP was significantly associated with cognitive decline, both 1 day (*p* = 0.001) and 1 month (*p* = 0.02) after surgery. With these findings, the authors emphasized the role of impaired cerebrovascular autoregulation in developing POCD, as hypertensive patients are often less adaptive to lower blood pressures. These results, however, should be interpreted with caution, since the study population was small (*n* = 45) and the history of antihypertensive drug treatment was not taken into consideration.

During 2009–2010, Panda et al. in a sample of 30 hypertensive and 30 non-hypertensive patients, aged 40–59 years, undergoing elective non-cardiac procedures under general anesthesia lasting ≥2 h observed a postoperative significant decline in cognitive function in both groups [31]. However, the POCD pattern differed between groups, based on Z-scores in a battery of 7 neuropsychological tests. Importantly, there was a noticeable difference in minimal intraoperative MAP, i.e., 93.8 mmHg vs. 68.6 mmHg (*p* < 0.001). The reader should bear in mind two major limitations of the study, including lack of adjustment for hypotensive events during surgery and a short observation period, lasting only 7 days.

Heyer et al. assessed the influence of intraoperative BP on early cognitive deficit in 183 patients undergoing cross-clamp open carotid endarterectomy under general anesthesia between 1995 and 2012 [32]. They found that patients whose intraoperative MAP was maintained ≥ 20% of baseline values, exhibited an 82% lower risk of POCD (*p* < 0.001). In their study, POCD was assessed by a local neuropsychologist using self-developed tests. Nevertheless, as the procedure itself is usually burdened with sudden changes in BP supply to the brain, causing possible disturbances in cerebral blood flow, higher MAP should be ensured to guarantee adequate perfusion.

Another interesting study was set up to verify the relationship between perioperative hemodynamic management and the risk of new-onset dementia [33]. The study was performed in 696 individuals averagely aged 84 years undergoing hip fracture surgery in either regional (8%) or general (92%) anesthesia. Within 6 or 12 months after surgery, dementia was diagnosed in 12% of patients using ICD-10 research criteria for dementia. Surprisingly, patients with MAP ≥ 130 mmHg at admission had over 5-times higher risk of dementia compared to subjects with MAP < 110 mmHg (*p* = 0.035). In addition, those whose MAP at admission was >100 mmHg had over 10-times higher risk of dementia compared to individuals with MAP < 80 mmHg (*p* = 0.009). This phenomenon can be explained by the fact that previous long-lasting arterial hypertension, which was diagnosed in 37% subjects preoperatively, might alter vascular autoregulation and made the cardiovascular system more susceptible to the effects of intraoperative BP drops. The results should be interpreted with caution because the assessment was performed in two different cohorts of patients hospitalized in 2005/2006 (*n* = 364) and 2009/2012 (*n* = 332).

Wang H et al. published the results of their research on the influence of controlled hypotension on postoperative cognitive function in 20 elderly spinal surgery patients, performed from 2013 to 2014 [34]. Controlled hypotension was defined as a MAP not less than 55 mmHg but no upper BP limit was set up. Importantly, intraoperative MAP was over 25 mmHg lower than the value recorded directly after induction of anesthesia (61.4 mmHg vs. 87.2 mmHg). The authors revealed that the crude score in MMSE was significantly lower on a postoperative day 1 and POCD (i.e., MMSE score at least 2 points lower than the preoperative one) occurred in 20% of subjects but that effect disappeared on day 7. The sample size was small and no effect of demographic of clinical data was examined.

Ni C et al. enrolled 78 orthopedic patients (mean age 70 years) undergoing total knee arthroplasty between 2014 and 2015, and screened them for POCD on days 1 and 6 after surgery [35]. The patients received regional anesthesia and no sedative agents were applied. POCD was diagnosed based on the battery of ISPOCD neuropsychometric tests. In subjects who developed POCD, a statistically significant decrease in MAP was observed after induction of anesthesia, with a mean drop of 22.24 mmHg compared to 13.22 mmHg in patients without POCD (*p* = 0.01).

A group of Turkish investigators performed a prospective, randomized clinical trial to compare cognitive function in 46 patients undergoing moderate hypotensive (i.e., MAP 60–70 mmHg) or normotensive anesthesia for endoscopic sinus surgery [36]. The results were published in 2005. There were no significant differences between the two groups in MMSE and the Visual Aural Digit Span Test (VADST) 2 h and 24 h postoperatively. Unfortunately, the study group was too small, BP drop was rather too low and the observation period too short to draw reasonable conclusions.

In 2015, Deiner et al. published their results on a study recruiting patients older than 65 years scheduled for major general, spine, urologic or thoracic surgery under general anesthesia [37]. POCD was defined based on a complex neuropsychological assessment with a battery of tests, MMSE and Confusion Assessment Method. Subjects with POCD were exposed to IOH on a similar extent as patients who did not develop cognitive decline (5 incidents in both groups, *p* = 0.78). Hypotension was defined as a number of incidents of MAP < 55 mmHg lasting for 5 min. Two limitations are crucial: significant impact of a type of anesthesia (total intravenous anesthesia, TIVA vs. volatile) and a small study group in a 3-month follow-up (*n* = 77).

In the next study, Chuan et al. explored a role of cerebrovascular autoregulation impairment in developing POCD in 140 subjects (mean age 68 years) undergoing abdominal, thoracic and vascular procedures using general anesthesia between 2014 and 2016 [38]. They found that defective cerebrovascular autoregulation (using tissue oxygenation index of dynamic autoregulation, TOx) was linked to the failure of cognitive recovery, assessed 3 days after surgery using the cognitive domain of the Postoperative Quality of Recovery Scale. The TOx in patients with cognitive recovery compared with those with impaired cognition was statistically significantly lower (0.06 vs. 0.18, *p* = 0.02). Those findings shed light on the role of the management of intraoperative BP to ensure adequate cerebral perfusion and cognitive performance in the postoperative period.

Finally, between 2014 and 2016, Langer et al. performed a randomized controlled trial in which 101 patients aged 80 ± 4 years were allocated into two groups [39]. The first group—the ‘target group’—had a personalized strategy of keeping MAP ≥ 90% of preoperative values, whereas the second group (the control one) had a liberal approach of intraoperative BP management. Despite having a higher intraoperative MAP (93 mmHg vs. 85 mmHg), no significant improvement was found in the prevalence of POCD after 3 months (11% vs. 7%, *p* = 0.5) in the study group. POCD was diagnosed by an experienced neuropsychologist using a battery of 7 tests. Some drawbacks should be taken into account in data interpretation, including relatively small sample size and a 24% drop-out rate for the neuropsychological testing after 3 months. Additionally, no standardized protocol for hemodynamic management was implemented.

Available studies on the association between intraoperative hypotension (IOH) or intraoperative blood pressure drop and postoperative cognitive decline (deficit) (POCD) are summarized in Table 5.

### 3.2. Postoperative Delirium

One of the first attempts to assess the impact of intraoperative hemodynamic parameters on the development of POD was made by Marcantonio et al. [40] who analyzed this issue in a group of 1341 patients over 50 years of age undergoing major elective non-cardiac surgeries. POD was diagnosed in only 9% of subjects, using a structured interview. There was no association between IOH and the development of delirium (POD vs. non-POD: 8% vs. 9%, *p* = 0.4). The duration of IOH also did not affect the number of POD episodes.

Over 10 years later, Tognoni et al. [41] identified IOH as one of the predictors of POD in 90 patients aged 74 ± 0.4 years undergoing urological procedures. In their study, hypotension was defined as a drop in systolic BP <90 mmHg requiring implementation of fluid therapy or infusion of catecholamines. POD, diagnosed by Confusion Assessment Method (CAM) algorithm, developed in 8.8% of patients, usually on postoperative day 1 and lasted for 3 ± 0.8 days. IOH occurred more frequently in the POD group (66% vs. 24%; *p* = 0.02); however, BP values were recorded in 15-min time intervals, what is a serious limitation in reliable concluding.

In a study published in 2011, Patti et al. [42] assessed 100 patients aged over 65 years undergoing colorectal oncological surgeries under general anesthesia. IOH was considered as a decrease in MAP <60 mmHg or the need for infusion of catecholamines. The screening for POD was performed twice a day with the CAM algorithm, and allowed to diagnose delirium in 18% of patients. It was found that IOH was a strong independent factor of POD (OR = 9.74; 95%CI 2.5–37.9, *p* = 0.001).

In 2015, Hirsch et al. [43] diagnosed POD with CAM in more than a half (i.e., 54%) of 540 patients aged 73 ± 6 years, who underwent major non-cardiac procedures. The authors revealed that IOH, defined as an absolute MAP drop <50 mmHg or relative decrease in MAP or systolic BP by more than 20, 30 or 40% from the baseline value, had had a significant impact on POD development (*p* > 0.05 for all). In addition, higher intraoperative variability of BP, calculated as a variance from all intraoperative BP measurements, was associated with a slightly higher risk of delirium (OR = 1.04; 95%CI 1.01–1.07, *p* = 0.008).

Wang J. et al. [44] investigated the impact of 12 perioperative variables on POD, that was assessed in 200 geriatric patients aged over 65 years who underwent elective orthopedic procedures. Seven factors have been identified as risk factors for POD, namely: age, type of anesthesia (general vs. regional), duration of surgery, intraoperative hypercapnia, IOH, altered preoperative mental state and postoperative sleep disorders. However, the authors did not provide the IOH diagnostic criteria, which is a serious limitation for drawing reliable conclusions.

In addition, in the elderly orthopedic population (i.e., *n* = 103, average age 82 ± 7 years), Wang NY. et al. [45] evaluated intraoperative BP as a function of mean intraoperative MAP (msMAP) and the percentage change in msMAP in relation to the baseline BP value. For each patient, the average msMAP was calculated as the area under the MAP curve formed by the 5-min recordings during surgery, divided by the length of surgery. Baseline MAP value was calculated as the mean of BP measurements from the moment of patient’s admission to the hospital until induction of anesthesia. The assessment for POD was performed on day 2 after surgery with CAM, what resulted in diagnosing delirium in 23% of patients. It was shown that in the group of subjects with msMAP <80 mmHg the increase of msMAP by 10 mmHg decreased the risk of POD (OR = 0.21, 95%CI 0.05–0.86, *p* = 0.03), whereas in the group of patients with msMAP ≥80 mmHg the increase of msMAP by 10 mmHg increased the risk of POD (OR = 2.34, 95%CI 1.11–4.94, *p* = 0.03).

Contrarily, in a study published in 2016, recruiting 480 patients aged 81 ± 6 years undergoing major non-cardiac surgery, it was found that IOH, defined as a decrease in baseline MAP >30%, had no impact on the frequency of POD episodes (5.8 vs. 6.1 episodes; *p* = 0.9) [46]. POD diagnosed with CAM occurred in 28.5% of individuals. The serious drawback of the study is the fact that baseline MAP was determined based on the measurements taken only 10-min before anesthesia induction.

Interestingly, secondary analysis of data from a study of van Grootven and colleagues [47] revealed that among 86 patients aged 80 ± 7 years undergoing orthopedic surgery, the lowest intraoperative diastolic BP was the protective factor for POD (OR = 0.92; 95%CI 0.85–0.99, *p* = 0.03) and the protective impact of the highest systolic BP was of a borderline significance (OR = 0.97; 95%CI 0.94–1.0; *p* = 0.06). The assessment of POD with CAM was performed in a complex manner on days 1, 3, 5 and 8 post-surgery. POD was diagnosed in 12.2%, 15.3%, 6.1% and 4.5% of subjects, respectively. This phenomenon is difficult to explain and was not discussed properly by the authors.

Soh et al. [48] investigated the issue of POD in 109 subjects aged over 60 years. IOH was defined as a decrease in MAP <80% of preoperative value or systolic BP < 90 mmHg, or absolute MAP <60 mmHg. IOH episodes were rare, however, were more frequent in those who developed POD (4 vs. 2 episodes; *p* = 0.01). The reader should bear in mind two major limitations of the study, including monitoring of BP in 15-min time intervals and the assessment of POD lasting only 48 h after surgery.

In the next study performed in the spinal surgery setting, Jiang et al. [49] retrospectively evaluated clinical charts of 451 patients aged 65 ± 18 years who underwent spinal surgery. POD, diagnosed with CAM was found in 9.3% of patients. Systolic BP <80 mmHg was considered as IOH. IOH was described by the authors as one of the main factors influencing the development of POD but the interpretation of statistics is rather not so convincing (i.e., OR = 7.5; 95%CI 0.18–17.93, *p* = 0.03).

In the study published in 2017 by Neerland et al. [33], no association between the lowest intraoperative MAP and POD was found among 696 elderly orthopedic patients, i.e., aged 85 years (IQR 78–89) with femoral neck fracture (POD vs. non-POD: 63 mmHg vs. 67 mmHg, *p* = 0.24). Screening for POD using CAM was performed once a day, except for the weekends, which should be taken into account in data interpretation.

In 2017, Guo et al. [50], basing on data from 385 patients with a mean age of 48 ± 13 years undergoing escharotomy, designed a predictive model to assess the probability of POD. One of the variables included in the model was IOH, defined as MAP drop <55 mmHg (OR = 27.07; 95%CI 7.13–102.83, *p* < 0.05). Patients with POD, representing 14.6% of the studied population had significantly more incidents of IOH (78% vs. 9%; *p* < 0.001). One ought to notice that the evaluation of POD using CAM was performed only once a day.

More recently, Langer et al. [39] performed a study covering 101 patients aged 80 ± 4 years scheduled to various non-cardiac surgeries. The authors investigated two strategies of the intraoperative BP control. In the first group, personalized management consisting of maintaining MAP ≥90% of preoperative value was applied, while in the second group, no target values were defined, leaving MAP values for the anesthesiologist’s decision. Interestingly, both strategies had similar and non-significant effect on the risk of POD (*p* = 0.2), which was observed in 9.9% of all patients using CAM-ICU for evaluation.

Additionally in 2019, in a group of 323 patients at a mean age of 60 years undergoing laryngectomy, POD by CAM occurred in 8.7% of them [51]. POD was more frequent in subjects who had IOH (*p* = 0.03). IOH was defined as BP lower than 70% of the baseline value lasting for more than 30 min.

For the study of Maheshwari et al. [52], aiming to assess the effect of IOH on POD, 1083 patients aged 62 ± 14 years undergoing organ transplantation (20%), gastrointestinal surgery (28%), orthopedic surgery (22%) or other non-cardiac surgery (30%) were recruited. Hypotension was considered as a MAP drop <65 mmHg. The assessment for delirium was performed twice a day with CAM-ICU. The delirium was found in 35% of individuals. It was shown that IOH was a significant risk factor for POD (HR = 1.11; 95%CI 1.03–1.20, *p* = 0.009). Moreover, the study demonstrated that POD was more frequent in patients with postoperative hypotension: the risk increased significantly with the decrease of the lowest postoperative MAP (HR = 1.12; 95%CI 1.04–1.20, *p* = 0.003).

In a small study covering 39 patients (median age 52 years, IQR 47–57) undergoing elective endovascular intracranial aneurysm treatment [53], the maximal intraoperative decrease of systolic BP was significantly higher in those who developed POD (33 mmHg vs. 25 mmHg, *p* = 0.002), but this effect was not confirmed in multivariate analysis (*p* = 0.5). POD was diagnosed in 35% of patients by using the CAM algorithm.

Finally, Radinovic et al. [54] in 277 orthopedic patients aged over 60 years aimed to assess the effects of a single episode of IOH, BP variations and heart rate changes on POD development. POD was evaluated using CAM with identification of its type (i.e., hypo- or hyperactive). POD was diagnosed in 53% of patients, 75% of which had the hypoactive type. Among patients who developed POD, lower BP values were observed compared to patients without POD, respectively: 128 mmHg vs. 122 mmHg (*p* = 0.001) for systolic, 78 mmHg vs. 76 mmHg (*p* = 0.01) for diastolic and 95 mmHg vs. 91 mmHg (*p* = 0.001) for mean BP. Although the authors failed to demonstrate a cut-off point for the MAP value below which the risk of POD increased, they showed that the likelihood of POD increased in patients with MAP interval of 75–80 mmHg. Interestingly, the lowest MAP had a slightly protective effect on the occurrence of any type of POD (OR = 0.94, 95%CI 0.89–0.99, *p* = 0.01), which is difficult to interpret. It must be underlined that BP values in the POD group did not meet the IOH criteria that are usually adopted in all previously mentioned studies. However, we may hypothesize that for selected patients (e.g., those with preoperative hypertension) even a decrease in MAP to 80 mmHg may cause clinically significant organ hypoperfusion. Unfortunately, this study lacked the evaluation of hemodynamic parameters in the postoperative period, which might help to explain those interesting but vague associations.

The above-mentioned studies on the association between intraoperative hypotension (IOH) or intraoperative blood pressure drop and postoperative delirium (POD) are summarized in Table 6.

## 4. Discussion

### 4.1. Cerebral Perfusion and Its Monitoring

IOH has deleterious impact on brain function due to decrease in cerebral blood flow (CBF) leading to tissue hypoperfusion. To protect the brain against injury, cerebral autoregulation maintains stable blood flow despite changes in systemic blood pressure (BP), which is typically observed within the mean arterial pressure (MAP) range of 60–150 mmHg [55]. These limits are not entirely fixed but can be modulated by sympathetic nervous activity, the vascular renin-angiotensin system and any factor (notably changes in arterial CO_2_ pressure) that decreases or increases CBF [55]. Noteworthy, in chronic hypertension the limits of autoregulation are usually shifted toward higher BP values. Physiological mechanisms protect the brain against ischemia caused by IOH or hypertension. However, vasoconstriction is much smaller than autoregulatory vasodilation. Consequently, much greater changes in CBF occur with IOH than with hypertension. Autoregulation is also impaired or absent in the elderly, in diffuse atherosclerosis, diabetes or uremia when endothelium is severely injured. Moreover, multiorgan failure or organ injury due to sepsis may also indirectly lead to brain injury. Patients in whom cerebral autoregulation is altered have a greater risk of cerebral ischemia if IOH occurs. This deleterious effect may be exaggerated by hypoxia, anemia and brain–blood barrier disruption [56,57].

Maintaining adequate CBF requires personalized hemodynamic treatment, basing on fluid regimen in fluid-responsive patients, inotropic support in subjects with compromised heart function and implementation of vasoactive agents in subjects with vasoplegia [58]. Goal-directed therapy is effective only if adequate methods of hemodynamic monitoring are applied [59]. These methods should be adjusted to individual patient’s needs and possible contraindications, taking into account preoperative risk of complications, the type and extent of surgery [60]. Treatment protocols use dynamic parameters (typically stroke volume variation or pulse pressure variation) to verify fluid responsiveness and stroke volume changes (ΔSV) to assess heart function. Considering BP monitoring during anesthesia, in high risk patients and in high risk procedures, invasive measurement is always advised. However, if non-invasive BP measurement is used, between-measurement time intervals should be shortened.

Intraoperative monitoring of brain function is rather limited and encompasses two main techniques, namely brain perfusion assessment with cerebral near-infrared spectroscopy (NIRS) or transcranial Doppler (TCD), and electroencephalography (EEG), bispectral index (BIS), qualitative EEG (i.e., patient state analyzer) or EEG-based entropy [61,62,63]. The effects of NIRS monitoring of brain oxygenation for reducing the occurrence of POCD are uncertain and there is also uncertainty as to whether active cerebral NIRS monitoring has an important effect on postoperative delirium [63]. Ultrasound-guided neuromonitoring can detect vasospasm, cerebral embolism, assess cerebral perfusion and helps in clinical decisions and early therapeutic interventions [62]. Current evidence suggests that EEG-guided anesthesia reduces the rate of awareness during total intravenous anesthesia but there is insufficient evidence to recommend it for preventing postoperative delirium or postoperative neurocognitive decline [61].

### 4.2. POCD

The studies that focused on the relationship between IOH and POCD are severely heterogeneous. Our comprehensive review has shown that POCD occurred from 9% to 47% of the investigated populations. The populations of all studies used in our analysis varied from 20 patients to 1218, who were aged from 40 to 84 years. POCD was investigated among subjects undergoing various non-cardiac procedures, including vascular (*n* = 1), laryngological (*n* = 1), neurosurgical (*n* = 2) and orthopedic (*n* = 3). In the remaining 6 studies, procedures were not limited to one specific surgical field. Importantly, 9 of the 13 studies analyzed patients undergoing general anesthesia, 2 of 13 included patients who received either general or regional anesthesia and the remaining two assessments included patients who received regional anesthesia only.

In most of the studies different methods of neuropsychological testing were implemented, including MMSE [29,34,35,36], various batteries of popular neuropsychometric tests (for example, Williams-Russo [28] used a battery of 10 tests, Panda [31] 7 tests and Moller [12] 4 different tests) and some self-developed ones (Heyer et al. [32]). Such diversity and lack of standardization impedes the drawing of reliable conclusions and does not allow to formulate recommendations for clinicians for several reasons. Firstly, tests of diverse type, design, sensitivity and specificity significantly bias POCD detection even within the same population. Additionally, the time at which the neurological state was evaluated was also not standardized across the publications, both in pre- and in post-operative periods. Since POCD can develop over days, weeks or even months after surgery [64], it is difficult to establish a proper time threshold at which neuropsychological testing would be the most efficient in detecting cognitive decline or clinically significant fluctuations. For instance, Cheng et al. [35] screened patients for POCD 6 days after surgery, whereas Langer et al. [39] tested patients 3 months post-procedure. Early evaluation (i.e., within the first weeks in the postoperative period) could miss patients who have not yet developed cognitive deficits, while a late evaluation (i.e., several months after surgery) could miss patients who have already recovered from POCD or mistakenly include those who developed new cognitive decline due to dementia. Therefore, in order to capture the entire cross section of individuals, tests should be implemented in a serial manner, and preoperative evaluation is a must to draw reliable conclusions. Indeed, some researchers screened patients for POCD at different time intervals, e.g., 1 week and 4 months (Wiliams-Russo [28]), 1 week and 3 months (Moller [12]), and 1 day and 1 month after surgery (Yocum [30]). Lastly, it is worth noting that implementation of stricter definitions of POCD and acknowledgment of only severe changes within tests would probably highlight more clinically significant cases of patients with POCD. However, all the included studies were focused on assessing statistical significance of changes in neuropsychological testing which could have led to an increased incidence of clinically meaningless POCD.

Another limitation of the research concerns the definition of IOH. The sole fact that different BP thresholds were applied appears to be a barrier for effective complex analysis. Only 6 out of 13 studies described IOH as either a direct value [28,34,36,37] or a relative BP drop [12,29]. Additionally, the definitions varied in their established minimal threshold for IOH duration, ranging from the very moment of IOH occurrence to even ≥30 min. The other research (i.e., 7 studies) did not set a threshold, instead choosing to monitor a minimal MAP [39,40,44] or approach the issue differently, like Langer et al. [39] who kept MAP within 90% of preoperative values. One has to acknowledge that up-to-now, no universal internationally recommended IOH definition has been set up. Nevertheless, the newest data suggests that even brief durations of MAP below 60–70 mmHg are harmful [4]. Unfortunately, only one study [36] used this threshold. Finally, BP measurement using oscillometric devices in the operating theatre could be biased in patients with atherosclerosis [65] who constitute a vast majority of elderly patients, and this drawback should be taken into account for reliable assessment of IOH. It seems reasonable that at least two measurements should be used to estimate the real exposure to low BP.

Sadly, none of the cited papers carries a high quality of evidence regarding the role of IOH in developing POCD. Seven out of 13 projects discovered an association between intraoperative hemodynamics and POCD [30,35,38]. However, the IOH threshold was introduced in only 1 of those 7 studies [43]. Chuan et al. [38] proved that impaired cerebral autoregulation prolongs postoperative cognitive recovery, which corresponds with Heyer’s study, in which patients who had their MAP kept within 20% of baseline values exhibited lower risk of developing POCD. Additionally, Yocum et al. [30] showed that hypertensive patients with a lower minimal intraoperative MAP are more prone to suffer from POCD. Moreover, Cheng et al. [35] noted a higher drop of post-induction MAP in patients with worse cognitive outcomes. If these results are combined with observations of Neerland et al. [33] who revealed that patients with higher preoperative blood pressures (MAP >100 mmHg) were more likely to present POCD than those with lower values (MAP <80 mmHg), it would indicate the necessity for a personalized treatment using patient-oriented hemodynamic monitoring rather than using rigidly adopted IOH thresholds.

### 4.3. POD

Our review revealed that studies focusing on the relationship between IOH and POD were also biased due to significant heterogeneity between included research protocols. POD occurred from 8% to 54% of individuals across included studies, and CAM or CAM-ICU algorithms were commonly used for screening of delirium. The assessment of POD subtypes was performed in only one study, in which 75% of diagnosed cases were hypoactive type [54]. This drawback sheds light on the precise assessment of POD. Also, the frequency of the screening importantly differed between the reviewed studies. For example, Wang X. [53] evaluated patients once a day, Maheshwari [52] twice a day, while Neerland [33] did not perform POD assessment on weekend days, and Soh [48] observed patients only up to 48 h after surgery. It must be remembered that current guidelines recommend that evaluation should be performed every 8 h or at least once per nurse shift throughout the hospitalization [66].

Out of the 17 POD studies included in our review, only 10 presented precise IOH criteria. In two studies [41,49] IOH was defined as an absolute decrease in SBP < 90 mmHg. In the next three articles, MAP < 55 mmHg [50], MAP < 60 mmHg [45] and MAP < 65 mmHg [52], respectively, were applied as the cut-off points for IOH. In another two studies, the definition of IOH as a percentage decrease of MAP by 30% compared to the baseline [46,51] was adopted. In the next three studies, mixed definitions were tested—both an absolute and relative decrease in MAP or SBP [40,43,48]. In a study published by Wang [51], BP must have dropped <70% of the baseline value for 30 min to recognize IOH. Patti et al. [42] the recognized IOH also in the case of long-term intraoperative use of catecholamines. In the remaining 7 studies the lowest intraoperative MAP, SBP or DBP [33,45,47,53,54] values were assessed or different strategies of intraoperative BP management were evaluated [39]. Furthermore, the authors applied various methods of BP monitoring: invasive and non-invasive, and additionally, used different time intervals for measurements, ranging between 1 and 15 min, which means that shorter episodes of hypotension could have been missed. The current evidence suggests a significant increase in the risk of perioperative complications even with a short-term decrease in SBP <1 00 mmHg [67]. Although maintaining proper BP values is not equivalent to ensuring proper perfusion in microcirculation, providing adequate BP is always the first step in cardiovascular optimization. The importance of individually adjusted therapy based on advanced, dynamic methods of hemodynamic monitoring was emphasized in the POQI [4] consensus and in recently published papers [68].

In a few studies the patients were monitored with bispectral index during anesthesia [46,48]. Monitoring the depth of anesthesia allows anesthesiologist to precisely and safely dose anesthetics, which may affect the incidence of POD and POCD [69]. Moreover, the studies differed in terms of the methods used to determine the initial BP value. In the majority of cases, measurements taken before the induction of anesthesia were considered as baseline BP, while they may not correspond to the standard BP profile of each patient [70]. Wang NY et al. [54], unlike other researchers, calculated the baseline BP basing on measurements taken from the patients’ admission to the emergency room till the induction of anesthesia, which may provide more reliable data. In two studies, the researchers used cerebral oximetry to monitor brain saturation, which enables the management of intraoperative hemodynamic parameters of cerebral autoregulation [39,48]. Wang NY et al. [45] demonstrated that the risk curve for POD was J-shaped, thus both intraoperative hypo- and hypertension could be harmful. Finally, the progression of mental deficiencies within elderly populations may be affected by BP control in the immediate postoperative period, which was considered in only one study [52].

### 4.4. Major Concerns for Further Investigations

Our review is probably the first attempt to systematize existing data of the relationship between IOH and cognitive impairment in the postoperative period. Although this review has shown that the knowledge we have is inconsistent and based on heterogeneous data, it has identified areas that need to be standardized in order to allow for future comparison of studies.

The vast majority of the studies included in the review were not designed to evaluate IOH and did not analyze hypotension as a risk factor among others specific to surgical procedures. For example, some procedures may predispose to significant hemodynamic disorders due to the patient’s forced position. Anesthesia in prone position for neurosurgical procedures within the CNS and spine has a negative effect on the cardiac index. Normal blood pressure in this group of patients is maintained due to high peripheral resistance (SVR). Anesthetic agents, by reducing SVR promote IOH with inadequate filling of the vascular bed. In addition, the pressure of the inferior vena cava in the prone position reduces the venous return which translates into a decrease in cardiac output [71]. Susceptibility to IOH is also due to the method of anesthesia and is particularly evident in neuraxial blockade; therefore, sub-analyses must be carried out taking into account the anesthesia method and its effect on hemodynamic disorders [72]. For example, patients undergoing escharotomy were initially at higher risk of cognitive impairment, therefore, demonstrating a link between hypotension and POCD in this group of patients can be extremely difficult and results that cannot be compared with other types of surgery.

We have not found any association between stricter IOH definitions and worse neurocognitive outcome. Future studies that will focus on this matter should adopt contemporary hypotension thresholds [4]; however, these may not be associated with higher occurrence of neurocognitive disorders due to population variability in cerebral autoregulation. We, therefore, would suggest a more direct approach in monitoring cerebral perfusion and implementing other methods, such as cerebral near-infrared spectroscopy [63]. This could be a step toward finding the true association between hypoperfusion and worse cognitive functioning.

Although, in terms of POD, all studies were based on DSMV-based delirium diagnostics, in terms of POCD, the studies differed from one another, and subsequent studies should also standardize the way POCD is diagnosed.

## 5. Conclusions

Available data are quite inconsistent and there is a paucity of high quality evidence convincing that IOH is a risk factor of postoperative cognitive impairment. Considerable heterogeneity between studies is the major limitation to set up reliable recommendations regarding intraoperative hemodynamic management to protect the brain against hypotension-related hypoperfusion. Further well-designed and effectively-performed research is needed to elucidate the true impact of intraoperative blood pressure variations on postoperative cognitive functioning.

## Figures and Tables

**Table 1:** Major determinants of intraoperative hypotension [1,2,3].

Patient Related	Procedure Related
Older age	Anesthesia induction with propofol
Male sex	Combined regional and general anesthesia
Low pre-induction systolic arterial blood pressure	Longer procedure
High American Society of Anesthesiologists (ASA) physical status (PS) class	Emergency procedure
Use of antihypertensive medications	Major procedure
Hypertension	Procedure in prone position

**Table 2:** Risk factors of postoperative neurocognitive disorder (POCD) [12,14,15,16,17,18,19,20,21,22].

Preoperative Factors	Intraoperative Factors
Older age (>60 years old)	Excessive depth of anesthesia
Pre-existing cognitive impairment	Extensive surgery
History of alcohol abuse	Type of surgery
Low educational level	Re-operation
APOE4 genotype	Hyperglycemia
Cardiovascular diseases	Pulmonary complications
Use of anticholinergic agents	

**Table 3:** Predisposing and triggering factors of delirium [23,24,25].

Risk Factors				Triggering Factors
Demography-Related	Comorbidities-Related	Surgery-Related	Anesthesia-Related	Postoperative Period-Related
Age > 70	Cognitive status	Aortic aneurysm surgery	Premedication with benzodiazepines	Inadequate pain control/inadequate opioid analgesia/drug toxicity
Male gender	Anemia	Thoracic surgery	American Society of Anesthesiology Physical Class ≥ 3	Sepsis
Education level	History of smoking	Abdominal surgery	Transfusions > 800 mL	Prolonged mechanical ventilation
Alcohol abuse	Hypertension	Hip replacement surgery	Fluid fasting time > 6 h	Hypoperfusion/Hypoxia
Malnutrition	Diabetes melitus		Medication (anticholinergic drugs, dezocine, meperidine)	
	Peripheral vascular disease			
	Heart failure			
	Polymorphism apoE			
	Obstructive sleep apnea			
	Prior postoperative delirium			
	Visual/hearing impairment			
	Functional impairment			

**Table 4:** Search strategy implemented in the study.

#1. PubMed search on 04/10/2020 using ‘MeSH terms’ and ‘best match’ strategy:
((“Neurocognitive Disorders”[Mesh]) AND “Hypotension”[Mesh]) AND “Surgical Procedures, Operative”[Mesh]) (*n* = 47) OR ((“Intraoperative Period”[Mesh]) AND “Hypotension”[Mesh]) AND “Treatment Outcome”[Mesh]) (*n* = 15) OR ((“Perioperative Period”[Mesh]) AND “Hypotension”[Mesh]) AND “Outcome Assessment, Health Care”[Mesh]) (*n* = 38) OR ((“Treatment Outcome”[Mesh]) AND “Intraoperative Period”[Mesh]) AND “Hypotension”[Mesh]) (*n* = 15) OR ((“Intraoperative Period”[Mesh]) AND “Blood Pressure”[Mesh]) AND “Neurobehavioral Manifestations”[Mesh] (*n* = 7)
#2. PubMed search on 04/11/2020 using ‘key words’ and ‘best match’ strategy:
((intraoperative hypotension) AND (postoperative period)) AND (delirium) (*n* = 13) OR (intraoperative hypotension) AND (cognitive defect) (*n* = 3) OR ((perioperative) AND (hypotension)) AND (cognitive function) (*n* = 20) OR ((intraoperative) AND (hypotension)) AND (cognitive impairment) (*n* = 23) OR (intraoperative blood pressure) AND (delirium) (*n* = 78) OR (intraoperative hypotension) AND (outcome) (*n* = 1486) OR ((surgery) AND (blood pressure)) AND (cognition) (*n* = 952) OR (postoperative cognitive dysfunction) AND (blood pressure) (*n* = 134) OR (hypotension) AND (postoperative cognitive dysfunction) (*n* = 75)
#3. Embase search on 04/15/2020 using ‘all fields’ algorithm:
(‘perioperative period’/exp OR ‘perioperative period’) AND (‘cognition’/exp OR cognition) AND (‘blood pressure’/exp OR ‘blood pressure’) (*n* = 187) OR ‘cognitive defect’ AND ‘intraoperative hypotension’ (*n* = 12) OR ‘hypotension’ AND ‘intraoperative period’ AND ‘cognition’ (*n* = 232) OR ‘hypotension’ AND ‘intraoperative period’ AND ‘disorders of higher cerebral function’ (*n* = 258) OR ‘treatment outcome’ AND ‘intraoperative hypotension’ (*n* = 157) OR ‘postoperative cognitive dysfunction’ AND ‘hypotension’ (*n* = 83)

MeSH—medical subject headings.

**Table 5:** Summary of studies on the association between intraoperative hypotension (IOH) or intraoperative blood pressure drop and postoperative cognitive decline (deficit) (POCD).

Author	Design of the Study	Study Population	Other Factors Associated with POCD Established by the Study (Only with *p* < 0.05)	IOH Definition	POCD Screening Method	Effects
Moller et al.,1998 [12]	Observational, prospective	1218 patients undergoing major abdominal, thoracic and orthopedic procedures with general anesthesia	Older age (OR 1.3) Second operation (OR 2.7) Respiratory Complications (OR 1.6) Longer duration of anesthesia (OR 1.1, *p* = 0.01) Infectious complication (OR 1.7, *p* = 0.04)	MAP drop for at least 60% from baseline for at least 30 min	Battery of neuropsychological tests 1 week and 3 months post-procedure	No effect
Wiliams-Russo et al., 1999 [28]	Randomized controlled	235 patients undergoing orthopedic procedures with epidural anesthesia	Older age	Two arms of controlled IOH, with a MAP of 45–55 mmHg or 55–75 mmHg	10 neuropsychologic tests after e.g., 1 week and after e.g., 4-months postoperatively	No effect
Boos et al., 2005 [29]	Observational, prospective	55 patients undergoing orthopedic, vascular, urologic and general procedures with general anesthesia (51%) and epidural anesthesia (49%)	Pre-existing cognitive impairment, defined as preoperative MMSE score (for each point of MMSE) (OR 0.41, *p* = 0.0038)	Systolic BP decrease above 30% of a baseline value, lasting more than 5 min or needing vasopressor or inotropic support	MMSE before, 24 h after surgery and on postoperative days 3 and 7	No effect
Yocum et al., 2009 [30]	Post-hoc cohort analysis	48 patients undergoing spine surgery with general anesthesia	Duration of anesthesia	Not defined, BP was recorded as a fraction of baseline MAP	A battery of 5 neuro-psychometric tests on postoperative day 1 and 1 month after surgery	Hypertensive subjects with a significant drop of MAP had cognitive decline
Panda et al., 2016 [31]	Observational, prospective	60 patients undergoing various non-cardiac procedures with general anesthesia	*-*	Not defined	Neuropsychological Test Battery on postoperative day 7	Postoperative cognitive decline pattern differed in patients with lower minimal MAP
Heyer et al., 2014 [32]	Observational	183 patients undergoing cross-clamp endarterectomy with general anesthesia	Low educational level (per year) (OR 1.16, *p* = 0.003) Diabetes mellitus (OR 2.73, *p* = 0.03) MAP kept ≥20% of baseline MAP (OR 0.18, *p* = 0.001)	Not defined	Self-developed battery of neuropsychometric tests applied preoperatively and 24 h postoperatively	Patients who had intraoperative MAP maintained ≥ 20% of baseline values exhibited a 82% lower risk of POCD
Neerland et al., 2017 [33]	Observational, prospective	696 patients undergoing orthopedic procedures with general anesthesia (8%) and epidural anesthesia (92%)	Older age (OR 8.8, *p* = 0.006) ASA group ≥ III (OR 3.5, *p* = 0.038) Vasopressor used during surgery (OR 3.5, p = 0.027) Postoperative MAP >100 mmHg (OR 10.6, *p* = 0.027) Delirium during hospitalization (OR 6.7, *p* = 0.001)	Not defined	ICD-10 research criteria for dementia applied within 6 or 12 months after surgery	Patients with MAP >100 mmHg had over 10-times higher risk of dementia compared to individuals with MAP <80 mmHg
Wang H et al., 2017 [34]	Randomized controlled	80 patients undergoing spine surgery with general anesthesia	*-*	Controlled hypotension was defined as a MAP not less than 55 mmHg, but there was no upper BP limit	MMSE preoperatively and on postoperative days 1 and 7	The crude score in MMSE was significantly lower on a postoperative day ‘1’ and POCD occurred in 20% of subjects
Ni C et al., 2015 [35]	Observational prospective	78 patients undergoing orthopedic procedures with epidural anesthesia	Older age (OR 1.2, *p* < 0.05) Lower average ScO2 after tourniquet deflation (OR 0.84, *p* < 0.05)	Not defined	Battery of neuropsychometric ISPOCD tests and MMSE, 1 day before surgery and on postoperative day 6	In subjects who developed POCD, a statistically significant decrease in MAP was observed after anesthesia induction
Sartacaoglu et al., 2005 [36]	Randomized controlled	46 patients undergoing endoscopic sinus surgery with general anesthesia	*-*	MAP 60–70 mmHg	MMSE and VADST after 2 and 24 h postoperatively	No effect
Deiner et al., 2015 [37]	Observational, retrospective	77 patients undergoing major non-cardiac procedures with TIVA or volatile anesthesia	MMSE in no-POCD vs. POCD patients (median) = 29 vs. 27.5 (*p* = 0.02)	MAP <55 mmHg lasting for at least 5 min	Battery of neuro-psychometric tests, MMSE and CAM within 30 days before and 3 months after surgery	No effect
Chuan et al., 2019 [38]	Observational, prospective	140 patients undergoing various non-cardiac procedures with	Older age Higher TOx	Not defined, MAP was maintained within a range of 75–90 mmHg	Cognitive domain of the Postoperative Quality of Recovery Scale preoperatively and on postoperative day 3	Defective cerebrovascular autoregulation was associated with the failure of cognitive recovery
Langer et al., 2019 [39]	Randomized controlled	101 patients undergoing various non-cardiac procedures with general anesthesia	*-*	Personalized treatment, study group (MAP within 90% of baseline) vs. control group	Neurocognitive evaluation performed by neuropsychiatrist with a battery of 7 tests preoperatively and 3 months after surgery	No effect

**Table 6:** Summary of studies on the association between intraoperative hypotension (IOH) or intraoperative blood pressure drop and postoperative delirium (POD).

Author	Design	Study Population	Other Factors Associated with POD Established by the Study (Only with *p* < 0.05)	IOH Definition	POD Screening Method	Effect
Marcantonio et al., 1998 [40]	Observational, retrospective	1341 patients; mixed major non-cardiac surgery	Age ≥ 70 year (OR 2.8) Cognitively impaired (OR 4.1) History of alcohol abuse (OR 2.8) Intrathoracic surgery (OR 3.0) Abdominal aneurysm surgery (OR = 9.1) Limited physical function (OR = 3.1) Lowest postoperative hematocrit ≤30% (OR = 1.7)	BP decline to 66% of preoperative baseline or <90 mm Hg requiring vasopressors or fluid resuscitation	CAM on postoperative days 2–5 or until the day before hospital discharge	No effect
Tognoni et al., 2010 [41]	Observational	90 patients; urology		Drop in systolic BP <90 mmHg, requiring implementation of fluid therapy or infusion of catecholamines	CAM daily for the first postoperative week	IOH occurred more frequently in patients with POD
Patti et al., 2011 [42]	Observational, prospective	100 patients; colorectal surgery	Preoperative albumin concentration (OR = 0.26) Alcohol abuse (OR = 5.76)	Decrease in MAP <60 mmHg or the need for catecholamines	CAM twice a day from the first postoperative day to hospital discharge or death	IOH was an independent risk factor for POD
Hirsch et al., 2015 [43]	Observational, prospective	540 patients; mixed major non-cardiac surgery	Age (OR 1.04) Gender (OR 1.71) Surgery duration (OR 1.24)	Absolute MAP drop <50 mmHg or relative decrease in MAP or systolic BP by more than 20, 30 or 40% from the baseline value	CAM preoperatively and on postoperative days 1–2	No effect, however higher intraoperative BP variability was a risk factor of POD (OR 1.04)
Van Grootven et al., 2016 [47]	Observational, prospective	86 patients; 15 orthopedic surgery (hip fracture)	Osteosynthesis surgery (OR 3.66)	Not defined	CAM, Delirium Index measured pre- and postoperatively (days 1, 3, 5, 8)	The lowest intraoperative diastolic BP was the protective factor for POD (OR = 0.92)
Wang J et al., 2015 [44]	Observational	200 patients; orthopedic surgery	Age (R = 0.04) Anesthesia type (R = 0.15) Surgical type (R = −0.04) Intraoperative hypercapnia (R = 0.12) Preoperative affective dysfunction (R= −0.13) Postoperative sleep disorders (R = 0.12) Hyperlipidemia (R= −0.09)	Not defined	CAM once a day for 7 post-operative days	IOH was associated with POD (R = −0.11)
Wang NY et al., 2015 [45]	Randomized controlled	103 patients; orthopedic surgery (hip fracture)	Age (OR = 1.09) Trial intervention (OR = 0.22) RBC transfusion (OR = 1.51)	Not defined	CAM on day ‘2’ post-surgery	Very high and very low MAP values were associated with significantly increased risk of POD (OR = 2.28)
Yang et al., 2016 [46]	Observational, retrospective	480 patients, mixed non-cardiac elective surgery	Hypertension (OR = 2.83) Tachycardia (OR = 2.74)	Decrease in baseline MAP >30%	Clinical observation within one week post-surgery, POD confirmed by a neurologist using CAM	No effect
Soh et al., 2016 [48]	Observational, prospective	109 patients; neurosurgery (major spinal surgery)		Decrease in MAP <80% of preoperative value or systolic BP <90 mmHg or absolute MAP <60 mmHg	CAM-ICU, ICDSC for 48 h after surgery	IOH episodes were more frequent in patients with POD
Jiang et al., 2017 [49]	Observational, retrospective	451 patients; neurosurgery (spine surgery)	Dezocine (OR = 6.68)	Drop in systolic BP <80 mmHg	Clinical assessment on preoperative day 1 and postoperative days 1, 2 and 3	No effect
Neerland et al., 2017 [33]	Observational, prospective	696 patients; orthopedic surgery (hip fracture)	NEADL <45 points (OR = 2.9) BMI <20 kg m^−2^ (OR = 4.5) ASA ≥ 3 (OR = 3.5) Blood transfusion ≥2 (OR = 3.4)	Not defined	CAM once a day preoperatively and until the fifth postoperative day (all) or until discharge (delirious patients), except for the weekends	No effect
Guo et al., 2017 [50]	Observational	385 patients; escharotomy	Age >50 years (OR = 7.68) Drinking >3 per week (OR = 19.34) ASA >II (OR = 302.48) Preoperative time >2 days (OR = 14.66) Number of previous escharotomy >2 (OR = 24.69) Operation time >180 min (OR = 81.86)	MAP <55 mmHg	CAM once a day for 5 postoperative days	Patients with POD had more incidents of IOH (OR = 27.07)
Langer et al., 2019 [48]	Randomized controlled	101 patients; mixed non-cardiac surgery		Not defined	CAM-ICU once a day for the first week post-operatively	No effect
Wang Y et al., 2019 [51]	Observational, prospective	323 patients; laryngectomy	Cancer stage (OR = 1.98)	BP lower than 70% of the baseline value lasting for >30 min	CAM once a day for 6 days post-operatively	IOH was more frequent in patients with POD
Maheshwari et al., 2019 [52]	Observational, retrospective	1083 patients; non-cardiac surgery		MAP <65 mmHg	CAM-ICU twice a day while patient remained in the ICU	IOH was associated with POD (HR = 1.11)
Wang X et al., 2019 [53]	Observational, prospective	39 patients; neurosurgery (intracranial aneurysm)	rSO_2_ desaturation score (OR = 1.002)	Not defined	CAM (frequency not specified)	The maximal intraoperative decrease of SBP was significantly higher in patients with POD
Radinovic et al., 2019 [54]	Observational, prospective	277 patients; orthopedic surgery		Not defined	CAM preoperatively and once a day for 7 post-operative days	Lower BP was observed in patients with POD
